# Improving Accessibility for Work Opportunities for Adults With Autism in an End-to-End Supported Workplace Program: Protocol for a Mixed Methods Cohort Study

**DOI:** 10.2196/60806

**Published:** 2025-05-01

**Authors:** Adam J Guastella, Lorna Hankin, Elizabeth Stratton, Nick Glozier, Elizabeth Pellicano, Vicki Gibbs

**Affiliations:** 1 Clinic for Autism and Neurodevelopmental Research, Brain and Mind Centre, Child Neurodevelopment and Mental Health Team, Children’s Hospital Westmead Clinical School Faculty of Medicine and Health University of Sydney Sydney Australia; 2 Central Clinical School Faculty of Medicine and Health University of Sydney Sydney Australia; 3 ARC Centre of Excellence for Children and Families over the Life Course University of Sydney Sydney Australia; 4 School of Education Macquarie University Sydney Australia; 5 Department of Clinical Educational and Health Psychology University College London London United Kingdom; 6 Autism Spectrum Australia Sydney Australia; 7 Children's Hospital Westmead Clinical School The University of Sydney Sydney Australia

**Keywords:** autism, neurodiversity, inclusive workplace, recruitment, job retention, accessibility, work opportunities

## Abstract

**Background:**

Adults with autism have a strong desire and need for employment that matches their strengths, skills, and interests. Yet, they are more likely to be un- or underemployed than their neurotypical peers. Facilitators to successful employment include positive attitudes toward and awareness of autism in the workplace, and provision of adequate support and accommodations, including mental health support. Despite previous workplace programs identifying the need for adapted recruitment and interviewing processes, awareness training, and ongoing employee support, there are no programs that specifically provide these supports and adaptations for employees with autism.

**Objective:**

In this study protocol, we describe a framework for a new end-to-end supported workplace program for adults with autism that encompasses 8 key areas to promote inclusive workplaces and improve recruitment and retention of employees with autism.

**Methods:**

Candidates with autism (n=15) will complete 2 workplace skills training sessions at the University of Sydney’s Brain and Mind Centre, before attending a tailored interview, onboarding session, and paid 12-week placement at consulting firm Ernst and Young (EY). EY managers and colleagues (n=30) will complete a half-day awareness and inclusion training program before supporting the adults with autism through their 12-week placements. Tailored mental health support will also be provided. This mixed methods cohort study will assess the change in the daily functioning and well-being of employees with autism, as well as the change in the managers’ and colleagues’ knowledge, attitude, and confidence in working with adults with autism following the training programs. These changes will be analyzed using repeated measures of ANOVAs.

**Results:**

Data collection for this study was completed in February 2022. As of November 2024, data analysis was in progress. This study is expected to be submitted for publication in June 2025.

**Conclusions:**

This study holds the potential to enhance the recruitment and retention of adults with autism, as well as their overall experience in the workplace.

**International Registered Report Identifier (IRRID):**

DERR1-10.2196/60806

## Introduction

### Background

Adults with autism have a strong desire and need for employment that matches their strengths, skills, and interests [1-3]. Yet, adults with autism are far less likely to be employed than their neurotypical counterparts [4-6], with global estimates of unemployment rates for adults with autism ranging up to 80% [7]. In Australia, the estimated rate of workplace participation for adults with autism is 42%, compared to 53% for individuals with other disabilities and 83% for those without a disability [8]. For adults with autism who do manage to secure employment, they are all too often in posts that are deemed unsuitable: either inconsistent with their skillset and abilities (mal-employment) or for which they are overqualified (underemployment) [9,10]. They can also find it difficult to maintain such employment [4-6].

A variety of barriers to successful employment outcomes for adults with autism have been identified at each stage of the recruitment and employment experience, including conventional hiring processes that are unsuited to candidates with autism, limited promotion of and requests for workplace accommodations, and a lack of employment-related and mental health supports [5,11,12]. Traditional job application and interviewing processes typically rely on in-vivo problem-solving skills and the navigation of complex social situations, which can pose unique challenges to some candidates with autism during recruitment [13-16]. Moreover, once in the workplace, employees with autism often report multiple environmental challenges, including navigating social expectations, experiencing aversive sensory stimuli, and stigmatizing attitudes from managers and coworkers [2,3].

Many organizations acknowledge a lack of awareness and knowledge of autism in the workplace [17], which has been linked to increased prejudice and stigmatizing attitudes [18]. For example, some employers perceive the costs of supporting employees with autism as outweighing any potential benefits [19,20]. This is despite evidence suggesting that employing adults with autism can provide substantial benefits, such as increased work ethic and quality of work, without incurring additional costs [21-24]. These studies underscore the need for more research to explore ways to reduce stigma and improve understanding of autistic people in the workplace.

Conversely, studies have identified a range of personal and environmental factors that can lead to successful employment outcomes for adults with autism [1,25,26]. Personal factors include having the knowledge to navigate recruitment and employment processes, work-related experience or skills, and insight into one’s strengths and challenges [25]. However, environmental factors have been identified as potentially having the greatest impact on successful employment for autistic people [26,27]. These factors include positive employer attitudes toward and awareness of autism, collaboration between the autistic employee and the workplace, and the provision of adequate support and accommodations, including mental health support [5,26,28,29].

While all autistic people experience autism in different ways and have different needs [30], there is some commonality in identified workplace supports. These include support in relation to executive skills, such as attention, planning, and problem solving [31]; navigating social environments within the workplace, which can be a source of both anxiety and confusion; and managing challenging sensory information [2]. There is also greater recognition that many autistic people experience co-occurring mental health concerns, such as depression and anxiety, associated with disability [32]. In fact, recent research found that mental ill health is a predictive factor in both vocational confidence and participation in adults with autism [18,33]. These findings suggest that mental health supports adapted to the needs of autistic people may be particularly important for workplace programs.

Research also points to the importance of the degree of “fit” between the job and the individual [25]. This job-matching process may need to take into account the task and social demands of the role, as well as individual skills, such as cognitive abilities, preferences, and sensory needs [2,3,17,25]. The better the job fit, the more likely the individual will succeed [25], highlighting the need for customized, tailored approaches that match individuals to specific roles.

The provision of appropriate support and accommodations can be dependent upon candidates disclosing their autism. However, many adults with autism are reluctant to disclose due to stigma and previous experiences of discrimination [34,35]. For adults with autism who do wish to disclose, the process is shrouded in tension, stress, and confusion as to how and when it should be done [35,36]. When adults with autism receive support to disclose, they are more likely to experience positive outcomes and receive the required accommodations [34,35,37]. Thus, any support program should support adults with autism in this nuanced process of disclosure during the selection phase.

A growing number of successful programs have been developed with the aim of recruiting and retaining neurodivergent employees [38-41]. Findings from these programs highlight the strengths and benefits that employees with autism can bring to an organization, such as diversity in thinking. In addition, they have identified the need for a range of accommodations, including building autism awareness in organizations seeking to employ autistic people, adapting recruitment and interviewing processes, and providing ongoing support to autistic job seekers and employees with autism once they are in their roles [38-41]. To date, however, programs have tended to focus on one aspect of these recommendations rather than providing comprehensive end-to-end support and adaptations for employees with autism and their employers.

### End-to-End Supported Workplace Program: A Framework

To improve workplace outcomes for adults with autism, there is a clear need for a holistic end-to-end approach, focusing on enhancing employers’ attitudes toward and understanding of autism, as well as providing adequate accommodations and services for candidates with autism throughout their recruitment and workplace experience [2,17,25,28]. Moreover, a tailored approach is required, matching work opportunities to an individual’s skills and strengths and providing workplace support based on individual needs, preferences, and experiences [3,25,28].

**Figure 1:**
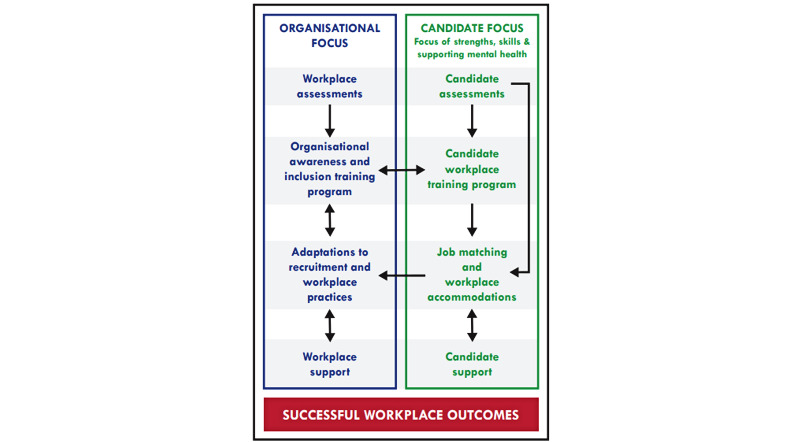
Supported workplace program framework.

Therefore, this study protocol proposes a framework that encompasses 8 key areas (Figure 1) to promote inclusive workplaces and enhance recruitment and retention of employees with autism before and during a 12-week placement at consulting firm Ernst and Young (EY). First, both the individual and the workplace are assessed to ascertain strengths and the supports needed to enable inclusive workplace practices. For the individual, this assessment includes their history of employment, strengths, skills, and interests, as well as expectations regarding job demands and practical requirements. For the workplace, it includes current workplace practices and safety within those practices, role and organizational demands, employer beliefs and attitudes, and existing presence and potential for inclusive support practices. Appropriate workplace adaptations can be recommended at this stage to enhance inclusive practices.

Next, training is provided, where required, to both workplace staff and candidates with autism. For the workplace, this training is designed to target knowledge and awareness, stigmatizing attitudes, and implementation of inclusive workplace practices. It also aims to influence adaptations to recruitment, onboarding, and employment processes across the organization. For candidates with autism, the training prepares and empowers them to navigate recruitment and workplace practices and roles, disclosure in the workplace, and the provision of workplace support and accommodations.

Candidates with autism are then matched with a role based on their preferences, strengths, and skills and provided with any necessary workplace accommodations during a 12-week placement.

Ongoing support is provided to both the organization and the candidates with autism throughout the program. The organization receives support during recruitment, onboarding, and the 12-week placement to continue disability and autism education and offer ongoing guidance on specific situations as they arise. Similarly, employees with autism receive support from mental health and autism workplace specialists throughout the placement, providing employment and mental health support, and tailored guidance addressing individual and contextual (workplace) challenges.

Drawing on the framework and available research, this study protocol describes a pilot program that encompasses an end-to-end 12-week supported work placement for adults with autism, based on individual needs, strengths, and preferences, as well as workplace training for candidates and workplace staff. Employers’ attitudes, knowledge, and confidence in supporting autistic people in the workplace before and after taking part in the program will also be explored. The results will provide researchers and employers with greater insight into how to optimize job fit and direct resources that can improve employment outcomes for adults with autism.

### Objectives

The study’s primary aims will be divided into 2 separate streams.

#### Stream 1: Adults With Autism

The primary objective of Stream 1 is to determine the feasibility of a 12-week supported work placement for autistic individuals based on individual needs, strengths, and preferences, with adaptations to traditional recruitment, interviewing, onboarding, and workplace processes. It is hypothesized that participating in the supported workplace program will result in improved workplace outcomes for adults with autism, as measured by job satisfaction, mental well-being, and general well-being. The secondary objective is to explore the facilitators and barriers (for example, autistic characteristics, social skills, mental health, and previous work experience) associated with successful employment (for example, increased job satisfaction and ongoing employment) in adults with autism.

#### Stream 2: Managers and Colleagues

Stream 2 investigates the knowledge and attitudes of—and support required by—the managers and colleagues of the adults with autism as they navigated the same processes. The primary objective in this stream is to determine the effectiveness of a face-to-face training workshop and 12-week supported workplace program in enhancing colleagues’ and managers’ knowledge, attitudes, and confidence in working with adults with autism. It is hypothesized that the program will increase employers’ knowledge and confidence in supporting people with autism in the workplace. A secondary objective explores the colleagues’ and managers’ current knowledge and attitudes toward hiring and managing autistic people and those living with disability more broadly.

## Methods

### Design Overview

The study uses an exploratory mixed methods cohort design with qualitative and quantitative data collected at multiple time points. Adult participants with autism will be screened for eligibility and invited to complete workplace skills training sessions at the University of Sydney’s Brain and Mind Centre (BMC), before attending an interview, an onboarding session, and a paid 12-week placement at EY in Sydney, Australia. EY managers and colleagues will complete a 2.5- to 3-hour training session, either face-to-face or online, before supporting the adults with autism through their 12-week placements.

The screening data of participants with autism will be collected via online questionnaires and face-to-face psychometric assessments, while baseline, midpoint, and postprogram data from adults with autism and baseline and postprogram data from colleagues and managers will be collected via online questionnaires. Postprogram semistructured qualitative interviews will be conducted with all participants via Zoom (Zoom Communications).

### Ethical Considerations

The protocol has received research ethics approval from Belberry Human Research Ethics Committee (HREC 2020-11-1229-A). Participants will be paid a standard wage for their internship hours but will not be paid for assessments conducted before or after the internship program for assessments or training. Participants will not be made aware whether their job is likely to be extended post internship. This is a discussion that will not be expected to happen and not until after the internship is complete.

### Setting

#### Partnering With EY

The 12-week supported workplace program will take place in partnership with EY. The roles available to adults with autism as part of the program will be based at EY’s head office in Sydney, Australia. Participants will also have the opportunity to work from home based on personal preference and to allow for ongoing COVID-19 restrictions, if applicable. Some roles are based on advertised positions. Others are new positions developed specifically to match the candidates’ strengths and skills, as indicated by the preprogram assessments and workplace skills training sessions. Face-to-face training workshops for EY managers and colleagues will be held at the EY head office and online.

#### Brain and Mind Centre, University of Sydney

The study will be implemented by researchers from the Clinic for Autism and Neurodevelopment Research (CAN Research) at the University of Sydney’s BMC. Candidates with autism will be predominantly recruited from previous participants of studies conducted by CAN Research. Preprogram screening assessments and workplace skills training sessions for adults with autism will take place at the BMC.

### Participants

#### Adults With Autism

Eligible participants will include those who have a current diagnosis of an autism spectrum condition and meet the criteria for an autism spectrum condition, as assessed at visit one by the Autism Diagnostic Observation Schedule—2nd Edition Module 4 (ADOS-2; Lord et al [42]), are fluent English speakers, can give written informed consent, and can work part- or full-time in Sydney for a period of 12 weeks. Participants will be excluded if they are not available for the study duration, have a drug or alcohol dependence, are younger than 18 years, or do not meet the criteria for an autism spectrum condition on the ADOS-2.

#### Managers and Colleagues

Eligible participants will include those who are available to manage or supervise at least one autistic adult working in their team at EY, have access to the internet, and can provide informed consent. Participants will be excluded if they are younger than 18 years or do not have reliable access to the internet.

### Recruitment

#### Adults With Autism

Adults with autism will be recruited from several sources, including a database of participants with autism who had previously taken part in studies at CAN Research and through external advertising. A job ad will also be posted on the online recruitment website SEEK to attract potential participants who may not yet be connected with autism support services.

All potential participants will be emailed a link to the online participant information sheet and consent form. Potential participants who express interest in the program via previous or current programs at CAN Research may have already completed the ADOS-2 and other executive function measures as part of those studies. They will, therefore, be screened for remaining eligibility criteria via a phone call from a researcher and an online questionnaire. Participants who express interest in the program after receiving information via the Clinic for Autism and Neurodevelopmental Research, ASPECT, or SEEK, will first be invited to the BMC to complete the ADOS-2 and other executive function measures. They will be screened for other eligibility criteria via an initial conversation with researchers at the BMC and an online questionnaire. Once identified, it will be up to EY to follow their standard human resource recruitment procedures to decide if they will hire candidates. The decision about which job is offered and whether the candidates accept the position will be entirely up to EY and the candidate. The rate of pay and the role will be provided to the candidate at the level expected for any person hired in that position. The decision about whether the internship will lead to a more permanent position is left up to EY at the end of the placement. Candidates are, however, not made aware that their position could be extended until completion of the placement to reduce the expectation of this outcome.

#### Managers and Colleagues

EY managers and colleagues will be recruited internally by the EY Diversity, Equality, and Inclusion (DE&I) Team. Managers and colleagues will be recruited from three tiers: senior managers and partners who led the teams involved in the program; managers working directly with the adults with autism, known as “counselors”; and colleagues working alongside the adults with autism, known as “buddies” (the counselor and buddy system operates within EY for all new starters). EY will identify specific teams to take part in the program depending on whether there is a vacant role available or the possibility of a newly created role within the team. Managers and colleagues are advised that participation is voluntary and has no impact on their employment relationship with EY. An internal EY email is sent with a link to an online participant information letter, consent form, and an online survey.

#### Workplace Skills Sessions

Once consent is obtained, eligible participants in both streams will complete baseline questionnaires and attend tailored workplace skills sessions.

#### Stream 1: Adults With Autism

The current literature has highlighted the requirements for, and benefits of, both recruitment and workplace support [1,9,25,28,38]. In response, we have developed two 2.5-hour workplace skills sessions for autistic participants to attend, covering resumes and interview skills, workplace scenarios, disclosure, and requesting accommodations. These will be held either at the BMC or online via Zoom.

Before the first session, participants will be asked to bring in a current copy of their CV to work on during the session. Facilitators will also provide a CV template and a completed example for guidance. Each section of the CV and its importance will be explained to the participants before individual feedback and guidance are provided. During the latter half of the first session, facilitators will share information on how to prepare for and take part in an interview. Participants will be offered the opportunity to prepare for and role-play common interview questions, to help reduce some of the anxiety experienced before and during interviews. We will also explain the specific adaptations EY is making to its interview process to prepare participants for their own interviews.

During the second session, facilitators will first share information about various aspects of the workplace that can sometimes pose challenges to employees with autism, including unwritten rules, communication and feedback, and ambiguous social etiquette. Participants will be given the opportunity to share examples of their experiences in previous workplaces and brainstorm possible solutions that they can use in the future. Finally, facilitators and participants will discuss options for full and partial disclosure and the type of accommodations available in the workplace. There will be an opportunity for role play and participants will be encouraged to complete an accommodations request form, which will be provided to EY.

#### Stream 2: Managers and Colleagues

Research has also highlighted the importance of providing workplace staff with training and guidance before taking part in a neurodiversity program [28,38]. To inform the training within this stream and to embed it within the context of this specific workplace, a sample of EY managers, colleagues, and employees with autism were previously surveyed about their attitudes and beliefs about autism [18]. EY managers and colleagues taking part in the program will attend a mandatory 3-hour training session before taking part in the supported workplace program. This can be attended in person or online, based on preference, availability, location, and COVID-19 restrictions. The training includes modules on autism awareness; disability inclusion; adapting recruiting, hiring, and onboarding practices; and tailored employee support and advancements. It is designed to provide attendees with a greater awareness of the unique barriers that employees with autism can face in the workplace and the support and opportunities that will make a difference in their experience and overall outcomes. Importantly, it offers real-world examples of challenges and solutions and will give attendees the opportunity to ask questions about, and role-play, specific scenarios that may occur in their teams.

### Job Matching

Adults with autism taking part in previous workplace programs indicated that they want placements that match their skills and interests [38]. Data from the executive function screening measures and baseline questionnaires will therefore be used to develop a personalized profile of strengths, skills, and preferences for each autistic candidate. This profile will include areas of interest; preferred working, communication, and feedback styles; executive function strengths; and preferred ways to manage potential challenges.

The profiles will be provided to EY’s DE&I Team, alongside CVs developed during the workplace skills sessions. The DE&I Team will use these documents to match the candidates’ strengths, skills, and experience with available roles within the participating teams, or to work with teams to create tailored roles for the duration of the 12 weeks. It is hoped that the better the job fit, the more likely the individual will have a successful workplace experience [5,25].

### Interviews

Once roles have been identified or created, the candidates with autism will be invited to interview. Interviews will be held in-person, with online options dependent on COVID-19 restrictions. Conventional interviewing practices can pose a number of challenges for candidates with autism [1,12]. To help overcome these barriers, standard interview processes may be adapted by, for example, reducing the interview duration; providing example questions in advance; allowing additional time and flexibility in how questions are answered; adapting the surroundings based on individual needs and preferences; and minimizing conventional interview requirements, such as eye contact and small talk. These changes will allow participants with autism a more supportive environment where they can focus on showcasing their strengths and skills rather than expending energy on interpreting ambiguous questions, dealing with sensory overload, or masking.

### Onboarding

Adaptions to onboarding will be recommended to make processes more accessible to candidates with autism, including reducing the time required for in-person and online onboarding to two half-days, delivering shorter sessions, and creating new written and graphic process documentation to allow for different learning styles. A buddy system will also be implemented during onboarding and throughout the 12-week placement to give employees with autism additional support and a designated go-to person to provide consistent guidance and feedback [5].

### Ongoing Support

Based on previous literature, showing that both employees with autism and their managers and colleagues benefit from ongoing support [5,38], the research team will provide weekly check-ins to all participants throughout the 12-week placement. The check-ins will be by email, phone, or Zoom calls depending on personal preferences. Both sets of participants will also be encouraged to call, text, or email researchers at other times should they need additional ongoing support or feedback. In addition, adults with autism will be offered mentoring sessions with an independent mental health-focused clinical psychologist and autism workplace specialist, who can provide more specialized workplace mentoring, mental health support, and career advice should it be required. Should this be required, support will be provided by a clinical psychologist working in the laboratory of the CAN Research but independently from this specific research study. This particular clinic has extensive experience working with the mental health needs of adults with autism and complex cases (eg, suicidality, acute mental health needs) will determine the level of support and whether withdrawal from the program is appropriate.

### Accommodations

Accommodations lists will be created by participants during the workplace skills sessions so that the workplace can implement preferences in advance of autistic participants starting in their roles. This will ensure that participants will arrive in an environment already set up to support their individual differences and unique needs [28]. By reducing the anxiety associated with disclosure and requesting accommodations [34,36], including being unsure of what is available and how to ask for it, autistic participants will be able to more confidently and comfortably settle into their roles [1,5]. The accommodation lists will also provide a greater awareness of how simple and inexpensive most accommodations can be [20]. Options for accommodations may include noise-canceling headphones; natural lighting; clothing preferences; quiet desks; supportive software; preferences for instructions, meetings, and performance reviews; and support with prioritizing and managing workflow.

### Primary and Secondary Outcome Measures

Measures, data sources, and time points are summarized in Tables 1 and 2. Measures have been selected that assess different aspects of the framework, which feed into the primary and secondary objectives for each stream.

**Table 1:** Summary of measures for adults with autism in the supported workplace program.

Measure		Screening	Preprogram	Midprogram (6 weeks)	Postprogram (12 weeks)
**Autism assessments**					
	Autism Diagnostic Observation Schedule, Second Edition (ADOS-2) [42]	✓			
	Ritvo Autism Asperger’s Diagnostic Scale 14-item screener (RAADS-14) [43]	✓			
**Drug and alcohol use**					
	Alcohol Use Disorders Identification Test (AUDIT) [44]	✓			
	Drug Use Disorders Identification Test (DUDIT) [45]	✓			
**Demographic factors**					
	Online questionnaire developed by the research team	✓			
**Social cognition**					
	Camouflaging Autistic Traits Questionnaire (CAT-Q) [46]	✓			
	Sensory Responsiveness Scale, Second Edition (SRS-2) [47]	✓			
**Behavioral factors**					
	Adult Repetitive Behaviour Questionnaire-2A (RBQ-2A) [48]	✓			
	Adult Sensory Profile (ASP) [49]	✓			
**Executive function**					
	Trail Making Test (TMT)	✓			
	Controlled Oral Word Test (COWAT)	✓			
	Wechsler Test of Adult Reading (WTAR) [50]	✓			
**Executive skills**					
	Online questionnaire developed by the research team	✓			
**Work preferences and experience**					
	Online questionnaire developed by the research team	✓			
**Daily functioning**					
	World Health Organization Quality of Life Scale (WHO-QOL BREF) [51]		✓	✓	✓
	World Health Organization Disability Assessment Schedule 2.0 (WHODAS) [52]		✓	✓	✓
**General well-being**					
	Bergen Insomnia Scale (BIS) [53]		✓	✓	✓
	Brief Resilience Scale (BRS) [54]		✓	✓	✓
	Coping Strategies Questionnaire [55]		✓	✓	✓
	Short online physical activities questionnaire developed by the research team		✓	✓	✓
**Mental well-being**					
	Depression Anxiety Stress Scales (DASS-21) [56]		✓	✓	✓
	World Health Organization–Five Well-Being Index (WHO-5) [57]		✓	✓	✓
**Job satisfaction**					
	Job Satisfaction Survey [58]			✓	✓
**Work-life balance**					
	Work-life Balance Survey [59]			✓	✓
**Workplace factors**					
	Everyday Discrimination Scale [60]			✓	✓
	An evidence-based question on bullying			✓	✓
	Job Content Questionnaire [61]			✓	✓
	Physical Work Environment Satisfaction Questionnaire [62]			✓	✓
**Inclusion and diversity**					
	Online questionnaire developed by the research team				✓
**Qualitative interview**					
	Questions developed by the research team, held via Zoom				✓

**Table 2:** Summary of measures for managers and colleagues in the supported workplace program.

Measures		Preprogram	Posttraining or postprogram
**Demographics and job factors**			
	Online questionnaire developed by the research team	✓	
**Knowledge of and attitudes toward autism**			
	Online questionnaire developed by the research team	✓	✓
**Social distance perceptions**			
	Adapted Bogardus Social Distance Scale [43]	✓	✓
**Views on working with autistic people**			
	Online questionnaire developed by the research team	✓	✓
**Self-efficacy**			
	Adapted Self-Efficacy Scale [63]	✓	✓
**Stress**			
	Kessler-10 (K-10) [64]	✓	✓
**Inclusion and diversity**			
	Online questionnaire developed by the research team	✓	✓

#### Adults With Autism

##### Screening Assessments

Autistic participants will be screened for eligibility and asked to provide demographic information. We will collect additional data on executive function; social cognition; behavioral traits; and work skills, preferences, and previous workplace experience to begin developing personalized job-matching profiles.

##### Preprogram

Following the screening, eligible autistic participants will complete an online survey to help finalize the personalized job-matching profiles and provide baseline data for pre-, mid-, and postprogram analysis. This will include questions on daily functioning, general, and mental well-being (see Table 1).

##### Midprogram

Autistic participants will complete an online survey six weeks into their 12-week placements. As well as daily functioning, general and mental well-being data will be collected on job satisfaction, work-life balance, and workplace factors, such as discrimination, bullying, job content, and physical environment (see Table 1).

##### Postprogram

At the end of the 12-week placement, participants will be reassessed on the measures given at baseline and mid-program, as well as completing a short online questionnaire designed by the research team to assess their perception and experience of inclusion and diversity in the workplace during their employment. Participants will then be invited to an individual semi-structured interview with a researcher via Zoom. This interview will explore their views and experiences of the program, including any potential barriers, and facilitators to a successful placement, as well as provide an opportunity to debrief and ask questions.

#### Managers and Colleagues

Managers and colleagues will be invited to take part in the program by the DE&I Team at EY and confirm they meet eligibility criteria during the informed consent process.

##### Preprogram

An online survey previously designed by the research team for another project sample in the same workplace [18] will be used to assess managers’ and colleagues’ attitudes toward and knowledge of autism, social distance perceptions, views on working with autistic people, self-efficacy, and stress. The survey also includes an assessment of attitudes toward diversity, inclusion, and corporate culture.

##### Postprogram

Managers and colleagues will complete the same online survey at the end of the 12-week placement. They will then be invited to an individual semi-structured interview with a researcher via Zoom. This interview will explore their experiences of supporting their autistic coworkers and any potential barriers and facilitators in doing so, as well as provide an opportunity to debrief and ask any questions.

### Analysis and Results

As this is an exploratory pilot program, 10-15 adults with autism will be recruited, alongside their managers and coworkers. Outcomes for both streams will be determined by:

- Quantitative assessments include completion and drop-out rates, percentage of components completed, job and workplace satisfaction, and employer attitudes and beliefs.
- Qualitative assessments that include employer and employee experiences and satisfaction with each component of the program. A per-protocol analysis will be conducted, whereby only complete data will be included in the analysis, where appropriate, imputation techniques will be used. As this is an exploratory study set in a real-world workplace, it is not feasible to control for confounding variables. There is no anticipated source of bias.

#### Quantitative

##### Stream 1: Adults With Autism

The primary objective in this stream is to determine the feasibility of a 12-week supported work placement for autistic individuals. The secondary objective is to explore the facilitators and barriers associated with successful employment in adults with autism. Data will be visually checked for data entry accuracy, and descriptive statistics will be used to check whether scores are within the valid range for each measure. Repeated measures ANOVAs will be used to assess temporal changes in the primary outcome measures taken at pre-, mid-, and postprogram time points, including changes to daily functioning, general and mental well-being, job and workplace satisfaction, and work-life balance.

Multiple linear regression will be used to explore intrinsic facilitators and barriers associated with successful employment for adults with autism. Predictor variables will be assessed for collinearity and then each predictor assessed with a univariate model. Significant predictors (*P*<.05) will be used to fit a multiple regression model to determine predictors of successful employment outcomes.

##### Stream 2: Managers and Colleagues

The primary objective in this stream is to determine the effectiveness of the training workshop and pilot program in enhancing colleagues’ and managers’ knowledge, attitudes, and confidence in working with adults with autism. A secondary objective explores the colleagues’ and managers’ current knowledge and attitudes toward hiring and managing candidates with autism.

Data will be visually checked for data entry accuracy, and descriptive statistics will be used to check whether scores are within the valid range for each measure. Repeated measures ANOVAs will be used to determine temporal changes to managers’ and colleagues’ knowledge, attitudes, and confidence in working with adults with autism, with primary outcome measures taken at pre-, mid-, and postprogram time points. Descriptive statistics will be used to explore and describe current knowledge and attitudes.

#### Qualitative

Audio recordings will be collected from participants in both streams via Zoom and transcribed verbatim. A semistructured interview schedule will be used to identify aspects of the program, such as adaptations to recruitment and workplace processes, which were perceived to be beneficial to the adults with autism, as well as areas that may need further improvements. The transcriptions will be deidentified, and each participant will be given a participant identification number as a pseudonym to protect anonymity.

#### Data Analysis

A total of 2 independent researchers will code the interview transcriptions using NVivo 11 (QSR International), using the Framework Method by Ritchie and Spencer [65] to analyze the data. The Framework Method is a type of thematic analysis commonly used to analyze semi-structured interviews that involve systematically reducing data into a matrix that allows for comparison between and within individual cases. The Framework Method allows for the structured identification of commonalities and differences in datasets. The method includes 5 stages: (1) transcription; (2) familiarization, beginning with transcription of the data; (3) categorization, where a thematic framework will be identified by the two coders. The coding will involve an iterative process as the codes are refined, and discrepancies resolved by a third investigator; (4) indexing, where key themes of direct quotes will be provided to demonstrate the richness of the themes. Further, subthemes within these key themes will provide a more in-depth understanding; and (5) charting, when themes will be tabulated so that the key and subthemes are easily identified.

## Results

The data collection for this study began in early 2021 and was completed in February 2022. As of November 2024, the analysis was in progress. This study is expected to be submitted for publication in June 2025.

## Discussion

### Principal Findings

This study seeks to explore the feasibility of a 12-week supported workplace program that includes adaptations and accommodations before and during the placement. It will also investigate employers’ attitudes, knowledge, and confidence in supporting employees with autism. Crucially, it builds on previous research and workplace programs to include workplace and candidate training sessions and ongoing tailored specialist support for both adults with autism and their managers and colleagues. It is anticipated that participating in the supported workplace program will result in improved workplace outcomes for adults with autism, as measured by job satisfaction, mental well-being, and general well-being. It is expected that the program will increase employers’ knowledge and confidence in supporting people with autism in the workplace.

### Comparison With Previous Work

There have been a number of supported workplace programs developed to improve employment for neurodiverse employees [38-41]. However, these programs tend to focus on a specific accommodation or only one element of employment, for example, adapting typical hiring and interviewing practices. This study incorporates the accommodations and supports identified in previous studies to propose an end-to-end framework. This framework aims to enhance recruitment and retention of employees with autism by providing support to both the employees and their managers before, during, and after the 12-week program.

The benefits of employment are well recognized. Improving workplace outcomes for adults with autism can offer significant benefits for the autistic community and the employment sector [21,22]. As well as economic independence, employment can offer adults with autism a sense of identity and accomplishment, improve quality of life, provide structure and opportunities for social interaction, and facilitate a contribution to society with less reliance on government funding [22,29]. Conversely, unemployment has been associated with lower life satisfaction and ill health [66].

Many adults with autism display myriad strengths that can contribute to the workforce, including excellent attention to detail, an expansive memory, an affinity for rules and guidelines, and the ability to analyze complex patterns in social and physical environments [23,24]. Workplace-specific research suggests adults with autism have higher levels of reliability, trustworthiness, attention to detail, and accuracy in visual tasks; expedient levels of concentration and memory; and lower levels of absenteeism than their counterparts [1]. By exploring the barriers, facilitators, and individual characteristics associated with successful employment, this study will provide employers with greater insight into where and how to direct resources in order to attract and retain talented candidates with autism. In turn, this will enhance the well-being and life satisfaction of adults with autism who want to work but have been unable to find roles that suit their strengths and skills. The findings from this study will be disseminated through publication in peer-reviewed journals.

### Strengths and Limitations

A growing number of programs with the aim of recruiting and retaining neurodivergent employees have been developed. However, these programs have tended to focus on providing a specific accommodation rather than end-to-end support. A strength of the current study is the implementation of an end-to-end framework to support employees with autism throughout the 12-week work placement. The mixed methods approach will allow for an in-depth analysis of the individual factors contributing to successful employment. We note, however, that the findings of this work may not be generalizable to all work contexts. EY primarily employs individuals with university-level education. They also have an established intention to increase neurodiverse employment throughout their workforce. Finally, we note that the individual assessments conducted on this sample size will provide limited opportunity to detect meaningful outcomes for predicting success. The completion of these measures is aimed at collecting pilot data for a much larger trial.

### Conclusion

The findings from this study will provide a greater understanding of factors relating to successful employment for adults with autism as well as provide insight into current attitudes in the workplace. Greater insights into current attitudes and knowledge of autism in the workplace, and the impact of a structured training program, will also help inform the development and implementation of much-needed similar training programs at other organizations. Former UN Secretary-General Ki-Moon [7] has called for companies to understand autistic individuals’ “unique and often exceptional skills, and to enable work environments where they can excel.” This study seeks to take another important step toward realizing that reality.

## Abbreviations

ADOS-2: Autism Diagnostic Observation Schedule–2nd Edition
BMC: Brain and Mind Centre
CAN Research: Clinic for Autism and Neurodevelopmental Research
DE&I: Diversity, Equality, and Inclusion
EY: Ernst and Young

## Data Availability

The datasets generated or analyzed during this study are not publicly available due to ethics considerations but are available from the corresponding author upon reasonable request.
